# Reactivation of autophagy by spermidine ameliorates the myopathic defects of collagen VI-null mice

**DOI:** 10.1080/15548627.2015.1108508

**Published:** 2015-11-13

**Authors:** Martina Chrisam, Marinella Pirozzi, Silvia Castagnaro, Bert Blaauw, Roman Polishchuck, Francesco Cecconi, Paolo Grumati, Paolo Bonaldo

**Affiliations:** 1 Department of Molecular Medicine; University of Padova; Padova, Italy; 2 Telethon Institute of Genetic and Medicine (TIGEM); Napoli, Italy; 3 Venetian Institute of Molecular Medicine (VIMM); Padova, Italy; 4 IRCCS Fondazione Santa Lucia; Rome, Italy; 5 Department of Biology; University of Rome Tor Vergata; Rome, Italy; 6 Danish Cancer Society Research Center; Copenhagen, Denmark; 7 Institute of Biochemistry II; Goethe University; Frankfurt am Main, Germany

**Keywords:** autophagy, collagen VI, muscular dystrophies, skeletal muscle, spermidine

## Abstract

Autophagy is a self-degradative process responsible for the clearance of damaged or unnecessary cellular components. We have previously found that persistence of dysfunctional organelles due to autophagy failure is a key event in the pathogenesis of COL6/collagen VI-related myopathies, and have demonstrated that reactivation of a proper autophagic flux rescues the muscle defects of *Col6a1*-null (*col6a1*^*−/−*^) mice. Here we show that treatment with spermidine, a naturally occurring nontoxic autophagy inducer, is beneficial for *col6a1*^*−/−*^ mice. Systemic administration of spermidine in *col6a1*^*−/−*^ mice reactivated autophagy in a dose-dependent manner, leading to a concurrent amelioration of the histological and ultrastructural muscle defects. The beneficial effects of spermidine, together with its being easy to administer and the lack of overt side effects, open the field for the design of novel nutraceutical strategies for the treatment of muscle diseases characterized by autophagy impairment.

## Abbreviations

BM: Bethlem myopathy
i.p.: intraperitoneal
Spd: spermidine
UCMD: Ullrich congenital muscular dystrophy

## Introduction

Macroautophagy (hereafter referred to as autophagy) is an evolutionarily conserved self-degradative process allowing cells and tissues to cope with several adverse stress conditions.^1-3^ Autophagy is constantly active at low rates in the whole organism, but various stress or pharmacological stimuli can induce its flux.^1,3^ In physiological conditions, autophagy plays a crucial role during development, cell differentiation and tissue remodeling, acts as a cell housekeeper by degrading damaged or unnecessary organelles, and allows the retrieval of metabolites under nutrient starvation.^3,4^ Age-related degeneration processes and neurodegenerative disorders, as well as liver, heart and muscle diseases, are only some of the pathological conditions where deregulated autophagy contributes to the pathophysiological defects, thus confirming the relevance of the autophagy-lysosome system for the homeostasis of organs and tissues.^3,5^

Skeletal muscle is an extremely plastic tissue, able to undergo extensive changes in response to physical exercise, dietary restriction and metabolic dysregulation.^6^ In this tissue, autophagy is essential for providing energy and eliminating harmful exhausted organelles.^6,7^ However, being a degradation process, autophagy needs to be finely regulated to maintain muscle mass and to ensure a proper removal of damaged organelles or protein aggregates. Indeed, alterations of the autophagic flux affect muscle homeostasis and also impinge on the body metabolic state.^7^ COL6/collagen VI-related myopathies represent a notable group of inherited muscle disorders where a link between dystrophic phenotype and autophagy flux impairment was first demonstrated. The key contribution of defective autophagy in the pathogenesis of muscle defects for these diseases is provided by studies in *Col6a1*-null (*col6a1*^−/−^) mice, in which abnormal signaling causes persistent AKT (v-akt murine thymoma viral oncogene homolog)-MTOR pathway activation and defective autophagosome formation in muscle fibers.^8^ Further studies in muscle biopsies of patients affected by Ullrich congenital muscular dystrophy (UCMD) and Bethem myopathy (BM), the 2 major human pathologies linked to mutations of COL6-encoding genes, confirm defective regulation of autophagy.^8^ Thereafter, defective autophagy induction has been reported in murine models for Duchenne muscular dystrophy and Emery-Dreyfuss muscular dystrophy, whereas increased autophagy flux contributes to the phenotype of *dy*^*3K*^*/dy*^*3K*^ mice, the animal model of MDC1A congenital muscular dystrophy.^5^ Notably, the autophagic defects are reversible and rescue of a proper autophagic flux by either genetic, pharmacological or dietary approaches leads to a strong amelioration of the muscle defects, a finding which led to a pilot clinical trial aimed at restoring autophagy in UCMD and BM patients through a low-protein diet approach (ClinicalTrials.gov Identifier NCT01438788).

Spermidine is a cationic polyamine naturally present in living cells at concentrations that decrease with aging. Spermidine supplementation to cell culture media or in vivo administration to entire animals prolongs life span in different model organisms, an effect which is linked to autophagy induction.^9,10^ Although the mechanisms by which spermidine acts are not yet fully understood, it has been shown that they involve a complex combination of acetylation and deacetylation events whose occurrence determines a shift in the acetylation pattern of the cell proteome without altering the total amount of acetylated proteins.^9^ Recently, the histone acetyltransferase EP300 has been described as the major target on which spermidine exerts an inhibitory activity, determining autophagy induction in vitro.^11^ Since in our previous studies we have found that reactivation of autophagy by either prolonged starvation or a low-protein diet is capable of rescuing the muscle pathology of *col6a1*^*−/−*^ mice,^8^ we evaluated in this myopathic model the efficacy of nutraceutical approaches able to forcibly induce autophagy. Here we show that spermidine administration by either addition to drinking water or intraperitoneal (i.p.) injection leads to a significant amelioration of the phenotype of *col6a1*^*−/−*^ mice. The ability to reactivate autophagy in COL6-deficient muscles, coupled with its low level of toxicity, points at spermidine as a nutraceutical with potential benefits for the treatment of these muscle diseases.

## Results

### Spermidine reactivates autophagy in *col6a1*^−/−^ muscles

To evaluate the therapeutic potential of spermidine in COL6-related muscle pathologies, we investigated wild-type and *col6a1*^*−/−*^ mice subjected to spermidine administration at different doses, durations and routes of administration. Spermidine was administered either by oral administration (*per os*), by addition to drinking water for 30 d, or i.p., by daily injection for 10 d. In both cases, 2 different doses were given (3 or 30 mM, *per os*; 5 or 50 mg/kg body weight, i.p.), in order to evaluate dose-dependent effects (**Table S1**).

**Figure 1 (see previous page).**
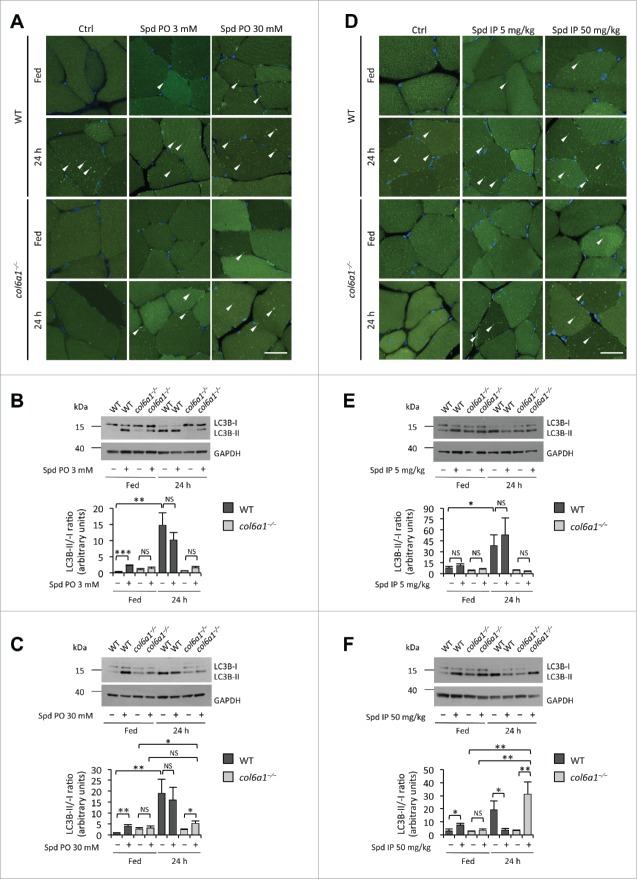
Spermidine induces autophagy in wild-type and COL6-deficient tibialis anterior muscles. (**A**) Detection of fluorescent puncta in cross sections of tibialis anterior muscles from untreated (Ctrl) and *per os* spermidine-treated (Spd PO) wild-type and *col6a1*^−/−^ mice bearing a GFP-LC3 reporter construct. Mice were analyzed under fed conditions or after 24 h fasting. White arrowheads point at some GFP-LC3-positive puncta. Nuclei were stained with Hoechst. Scale bar: 25 μm. (**B**, **C**) Western blot analysis of LC3B lipidation in protein extracts from tibialis anterior muscles of untreated (–) and *per os* spermidine-treated (+) wild-type and *col6a1*^−/−^ mice. Densitometric quantifications of LC3B-II *vs* LC3B-I ratio, as determined by at least 3 independent western blot experiments, are shown in the lower panels (*n* = 3 or 4; *, *P*< 0.05; **, *P* < 0.01; ***, *P* < 0.001; NS, not significant). (**D**) Detection of fluorescent puncta in cross sections of tibialis anterior muscles from untreated (Ctrl) and i.p. spermidine-treated (Spd IP) wild-type and *col6a1*^−/−^ mice. Mice were analyzed under fed conditions or after 24 h fasting. White arrowheads point at some GFP-LC3-positive puncta. Nuclei were stained with Hoechst. Scale bar: 25 μm. (**E**, **F**) Western blot analysis of LC3B lipidation in protein extracts from tibialis anterior muscles of untreated (–) and i.p. spermidine-treated (+) wild-type and *col6a1*^−/−^ mice. Densitometric quantifications of LC3B-II *vs* LC3B-I ratio, as determined by at least 3 independent western blot experiments, are shown in the lower panels (*n* = 3 or 4; *, *P* < 0.05; **, *P* < 0.01; ***, *P* < 0.001; NS, not significant). 24 h, twenty-four hour-long starvation; Ctrl, untreated control condition; Spd IP, spermidine i.p.; Spd PO, spermidine *per os*; WT, wild-type.

*Per os* administration of spermidine at both 3 mM and 30 mM was effective in stimulating autophagy in skeletal muscles. Fluorescence microscope analysis of tibialis anterior showed several GFP-LC3 puncta in wild-type animals after 30 d spermidine treatment (**Fig. 1A**). Autophagy induction was further confirmed by immunoblotting analysis of cleavage and lipidation of MAP1LC3B/LC3B (microtubule-associated protein 1 light chain 3 β), quantified both as LC3B-II/-I and LC3B-II/GAPDH ratios, which was increased at both low- and high-dose spermidine treatment (**Fig. 1B and C**; **Fig. S1A and B**). Of note, these treatments did not exert any overt change in the response to 24 h starvation in wild-type mice (**Fig. 1A-C**; **Fig. S1A and B**). In agreement with previous work,^8^ *col6a1*^*−/−*^ tibialis anterior displayed a markedly decreased autophagy in the 24-h fasting condition, when compared to the corresponding wild-type muscles. Interestingly, spermidine administration induced autophagy in *col6a1*^*−/−*^ muscles. Indeed, fluorescence microscopy showed the presence of GFP-LC3 puncta in tibialis anterior of spermidine-treated *col6a1*^*−/−*^ animals, which were clearly evident in mice treated with 30 mM spermidine and subjected to 24-h starvation (**Fig. 1A**). Immunoblotting analysis of tibialis anterior extracts confirmed a significant increase of LC3B-II/-I and LC3B-II/GAPDH ratios in fasted *col6a1*^*−/−*^ mice treated with 30 mM spermidine, when compared to fasted *col6a1*^*−/−*^ mice without spermidine, whereas no significant change in LC3B-II/-I was detected in *col6a1*^*−/−*^ mice maintained under fed conditions or treated with the lower dose of spermidine (**Fig. 1B and C**; **Fig. S2A and B**). Interestingly, the LC3B-II/GAPDH ratio was instead significantly increased in fed *col6a1*^*−/−*^ mice following a low dose *per os* treatment (**Fig. S1A**). In order to further clarify the status of the autophagy machinery in the skeletal muscle of fed wild-type and *col6a1*^*−/−*^ mice subjected to *per os* spermidine treatment at different doses, we analyzed by immunoblotting the protein levels of RAB7, a small Ras-like GTPase with an essential role in lysosomal biogenesis and in the late phases of endosome and autophagosome fusion with lysosomes,^12-13^ and GABARAP, a yeast Atg8 ortholog playing a key role in the final stages of autophagosome biogenesis.^14^ Both the lipidated form of GABARAP (GABARAP-II) and RAB7 were increased in a dose-dependent fashion following spermidine treatment not only in wild-type but also in *col6a1*^*−/−*^ mice. These data indicate an increased rate of autophagosome and autolysosome formation, respectively, and are consistent with autophagy induction in fed animals of both genotypes (**Fig. S2A-C**). Altogether, these findings indicate that spermidine supplementation in drinking water for 30 d, promptly induces autophagy in wild-type muscles and also affects the autophagic process of *col6a1*^*−/−*^ muscles.

In order to evaluate more precisely the effects of spermidine in inducing autophagy in skeletal muscles, we subjected wild-type and *col6a1*^*−/−*^ mice to daily i.p. injection with spermidine for 10 d (**Table S1**). Spermidine i.p. treatment at 5 mg/kg displayed no significant effects on fluorescent GFP-LC3 puncta and LC3B lipidation, both in wild-type and *col6a1*^*−/−*^ tibialis anterior (**Fig. 1D and E**; **Fig. S1C**). Slightly increased protein levels of GABARAP-II, but not RAB7, were detected in wild-type and *col6a1*^*−/−*^ tibialis anterior (**Fig. S2D-F**), further confirming that this dosage is unable to elicit a robust upregulation of autophagy. Conversely, spermidine i.p. treatment at 50 mg/kg triggered not only GFP-LC3 puncta formation and LC3B lipidation (**Fig. 1D and F**; **Fig. S1D**), but also a very strong increase in RAB7 and GABARAP-II protein levels (**Fig. S2D-G and I**) in muscles of wild-type mice maintained under fed conditions. Autophagy induction in this condition was further confirmed by the strong accumulation of the autophagosome markers LC3B-II and SQSTM1 (sequestosome 1) in tibialis anterior of wild-type mice treated simultaneously with spermidine i.p. 50 mg/kg and colchicine (**Fig. S2G-K**), an inhibitor of the late stages of autophagosome maturation and fusion with lysosome that allows investigating the autophagic flux in skeletal muscle.^15^ Consistently with the role of RAB7 in the intracellular vesicle trafficking and in the fusion of autophagosomes with lysosomes, this protein displayed unaltered levels in spermidine plus colchicine-treated animals with respect to untreated control conditions (**Fig. S2G-I**). Unexpectedly, i.p. treatment with spermidine at 50 mg/kg combined with 24 h starvation led to decreased LC3B lipidation in wild-type muscles although autophagic vacuoles were clearly detectable (**Fig. 1D and F**; **Fig. S1D**). This latter finding may be explained by the intensive and prolonged autophagy induction in this condition, which would lead to the consumption of LC3B protein via autophagosome-lysosome fusion and degradation.

Notably, treatment with spermidine at 50 mg/kg for 10 d led to an overt induction of autophagy in tibialis anterior of 24-h-fasted *col6a1*^*−/−*^ mice, as indicated by the presence of a large number of GFP-LC3 puncta and by the conspicuous increase in LC3B lipidation (**Fig. 1D and F**; **Fig. S1C**). In fed conditions, autophagy appeared similarly induced in muscles from *col6a1*^*−/−*^ mice subjected to i.p. treatment with spermidine at 50 mg/kg, based on the increased presence of GFP-LC3 puncta (**Fig. 1D**) and on the higher levels of RAB7 and GABARAP-II (**Fig. S2D to F**). Moreover, combined treatment with colchicine and i.p. 50 mg/kg spermidine led to a significant accumulation of SQSTM1 and the lipidated form of LC3B in tibialis anterior of *col6a1*^*−/−*^ mice. Of note, differently from what we observed for wild-type mice, colchicine treatment alone did not determine an accumulation of any of these autophagic markers in muscles from *col6a1*^*−/−*^ mice (**Fig. S2G-K**). This evidence further confirms the autophagy impairment of COL6-deficient muscles, and represents an unambiguous proof of the concept that spermidine is capable of reactivating autophagy in muscles of *col6a1*^*−/−*^ mice.

Altogether, these data indicate that spermidine is able to induce autophagy in skeletal muscle in a dose-dependent manner and is also effective in reactivating autophagy in *col6a1*^*−/−*^ muscles.

### Spermidine supplementation recovers the histological and ultrastructural defects of *col6a1*^−/−^ muscles

**Figure 2 (see previous page).**
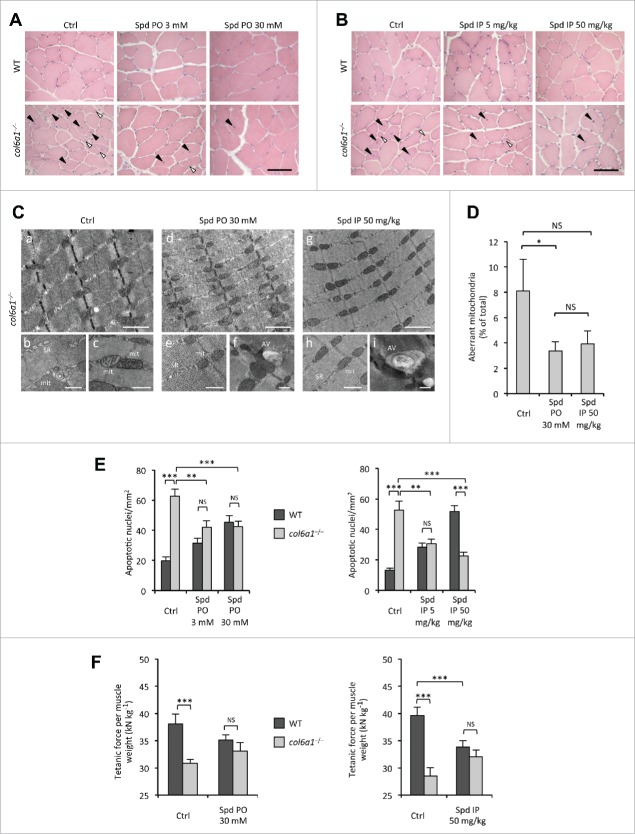
Spermidine treatment ameliorates the myopathic phenotype of *col6a1*^−/−^ mice. (**A**, **B**) Haematoxylin-eosin staining of cross sections of tibialis anterior muscles from untreated (Ctrl) and *per os* (A) or i.p. (**B**) spermidine-treated wild-type and *col6a1*^−/−^ mice. In *col6a1*^−/−^ mice, centrally nucleated fibers (black arrowheads) and atrophic fibers (white arrowheads) appear reduced after spermidine treatment. Scale bar: 50 μm. (**C**) Representative electron micrographs of diaphragm thin sections from untreated (a to c) and *per os* (d to f) or i.p. (g to i) spermidine-treated *col6a1*^−/−^ mice. Low-power views (upper panels) reveal improvement of muscle ultrastructure following spermidine treatment. High-power magnifications (lower panels) show dilated sarcoplasmic reticulum (SR) and swollen mitochondria (mit) in untreated *col6a1*^−/−^ mice (b,c), and the presence of autophagic vacuoles (AV) in spermidine-treated *col6a1*^−/−^ mice (f,i) together with a marked amelioration of organelle morphology (e,h). Scale bar: 1 μm (upper panels) or 500 nm (lower panels). (**D**) Percentage of morphologically altered mitochondria in the diaphragm of untreated (Ctrl) and spermidine-treated (PO 30, *per* os 30 mM; IP 50, i.p. 50 mg/kg) *col6a1*^*−/−*^ mice (*n* = 3 or 4; *P*< 0.05; NS, not significant.) (**E**) Quantification of apoptosis by TUNEL assay in diaphragm muscles of untreated (Ctrl) and *per os* (left panel) or i.p. (right panel) spermidine-treated wild-type and *col6a1*^−/−^ mice (*n* = 3, each group; **, *P* < 0.01; ***, *P* < 0.001). (**F**) *In vivo* tetanic force measurements in gastrocnemius muscle of untreated (Ctrl) and *per os* or i.p. spermidine-treated wild-type and *col6a1*^*−/−*^ mice. The histograms report the normalized strength at a stimulation frequency of 100 Hz (*n* = 5, each group; ***, *P*<0.001; NS, not significant). Ctrl, untreated control condition; Spd IP, spermidine i.p.; Spd PO, spermidine *per os*. WT, wild-type.

To assess whether spermidine administration has any effect on the structural defects of COL6-deficient myopathic mice, we performed histological analysis of tibialis anterior muscles from wild-type and *col6a1*^−/−^ mice subjected to the different protocols of spermidine treatment. In wild-type mice, treatment with spermidine did not cause any sign of histological changes in muscle architecture at the tested doses and routes of administration (**Fig. 2A and B**). As expected,^16^ under untreated conditions *col6a1*^*−/−*^ tibialis anterior displayed an overt myopathic phenotype, with centrally nucleated myofibers, small atrophic fibers and giant hypertrophic ones. Notably, both i.p. and *per os* spermidine treatments led to a noticeable amelioration of the myopathic defects. The effects of spermidine seemed to correlate with the dose, as *col6a1*^*−/−*^ mice treated with the high doses displayed improved muscle histology (**Fig. 2A and B**). Indeed, 3 mM *per os* and 5 mg/kg i.p. spermidine treatments led to a partial rescue of histological alterations in *col6a1*^*−/−*^ mice and, albeit less extended, the myopathic defects were still present, with some atrophic and degenerating myofibers (**Fig. 2A and B**). However, *col6a1*^*−/−*^ animals displayed markedly improved muscle morphology both after 30 mM *per os* and 50 mg/kg i.p. spermidine treatment, with barely detectable signs of myofiber degeneration, more homogeneous myofiber size and rare atrophic fibers (**Fig. 2A and B**). Some centrally nucleated fibers were present in treated animals, consistent with myofiber regeneration (**Fig. 2A and B**).

A well-known feature of *col6a1*^*−/−*^ muscles is the presence of altered organelles, in particular swollen mitochondria and dilated sarcoplasmic reticulum.^8,17,18^ Histological staining for SDH/succinate dehydrogenase, a mitochondrial marker, showed a more homogeneous labeling, pointing at an amelioration of the mitochondrial network in tibialis anterior of *col6a1*^*−/−*^ animals treated with higher spermidine doses (**Fig. S3**). Transmission electron microscopy of diaphragm ultrathin sections revealed a marked amelioration of sarcoplasmic reticulum and mitochondria ultrastructure in myofibers of spermidine-treated *col6a1*^*−/−*^ mice (**Fig. 2C**). Morphometric analysis confirmed a decreased percentage of aberrant mitochondria in muscles of spermidine-treated *col6a1*^*−/−*^ animals, which was significant after 30 mM *per os* treatment (*P* = 0.039) whereas it almost reached significance after 50 mg/kg intraperitoneal treatment (*P* = 0.077) (**Fig. 2D**). Furthermore, electron microscopy confirmed the presence of several autophagic vacuoles in spermidine-treated *col6a1*^*−/−*^ animals (**Fig. 2C**), consistently with the elimination of altered mitochondria through spermidine-induced autophagy.

Altogether, these data indicate that spermidine-mediated reactivation of autophagy ameliorates the myopathic defects of *col6a1*^*−/−*^ animals, with a significant decrease of swollen mitochondria and a marked improvement of muscle structure.

### Spermidine ameliorates the functional deficits of *col6a1*^−/−^ muscles

Given the noticeable improvement of muscle structural defects, we next investigated whether the tested doses of spermidine were also capable of ameliorating the deterioration in other muscle features. A well-established feature of *col6a1*^*−/−*^ mice and of UCMD and BM patients is the increased incidence of apoptotic myofibers in skeletal muscles.^8,17^ TUNEL analysis for diaphragm revealed that spermidine administration elicited a strongly decreased incidence of apoptotic myofibers in *col6a1*^*−/−*^ mice. Indeed, both *per os* and i.p. treatments led to a significant decrease in the number of apoptotic myonuclei in *col6a1*^*−/−*^ diaphragms, and this effect was displayed at all the tested doses of spermidine (**Fig. 2E**). The decrease appeared stronger after i.p. treatment, where the effect positively correlated with the dose. However, *per os* treatment also displayed a clear effect, with about 35% decrease in the number of apoptotic myonuclei at both low and high doses (**Fig. 2E**). Conversely, wild-type animals displayed an increased number of TUNEL-positive myonuclei directly correlating with the dose both after i.p. and *per os* treatment (**Fig. 2E**), likely due to the excessive induction of autophagy elicited by spermidine in wild-type muscles.

We have previously demonstrated that under physiological conditions *col6a1*^*−/−*^ mice develop significant less strength than wild-type mice.^8^ Therefore, we performed in vivo tetanic force measurements on gastrocnemius muscles of wild-type and *col6a1*^*−/−*^ mice under untreated conditions and following spermidine administration. Both *per os* and i.p. spermidine treatment led to a positive trend in normalized force in *col6a1*^*−/−*^ mice (**Fig. S4**), although the values obtained at a tetanic stimulation frequency of 100 Hz did not reach a statistically significant difference when compared to untreated animals (*P* = 0.138, *per os* treatment; *P* = 0.163, i.p. treatment). Of note, while the difference in the normalized force developed by wild-type and *col6a1*^*−/−*^ mice under standard conditions was highly significant, animals of the 2 genotypes displayed similar normalized force values after spermidine treatment (**Fig. 2F**; **Fig. S4**). As for apoptosis, high-dose i.p. spermidine administration caused a force decline in wild-type mice, likely due to the excessive autophagy induction elicited in these animals (**Fig. 2F**; **Fig. S4B**) as we previously observed in wild-type mice subjected to low-protein diet.^8^

Altogether, these data indicate that spermidine induction of autophagy recovers the apoptotic defects of *col6a1*^*−/−*^ animals, with also a positive trend in their muscle strength.

### Spermidine effects involve modulation of the AKT signaling pathway

**Figure 3.**
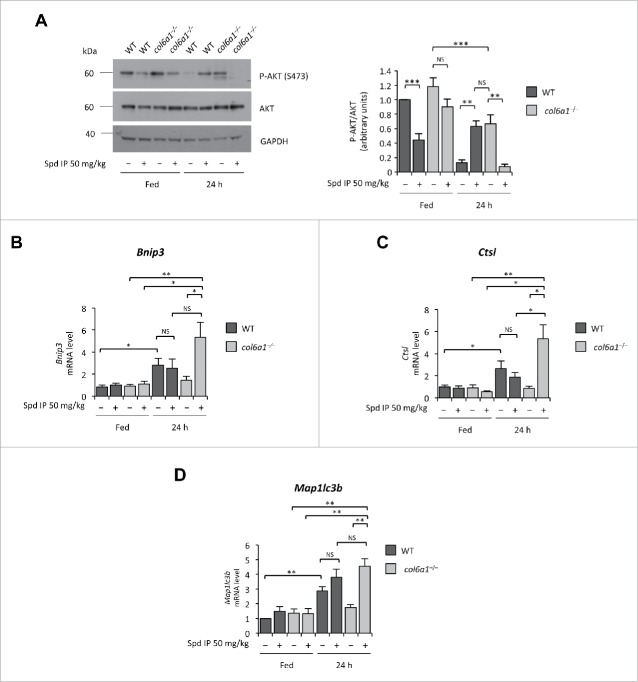
Spermidine modulates the AKT-FOXO axis in skeletal muscle. (**A**) Western blot analysis of AKT phosphorylation in protein extracts from tibialis anterior muscles of untreated (–) and i.p. spermidine-treated (+) wild-type and *col6a1*^−/−^ mice. Densitometric quantifications of P-AKT *vs* AKT, as determined by 3 independent western blot experiments, are shown on the right panel (*n* = 3; **, *P*< 0.01; ***, *P* < 0.001). (**B**–**E**) Real-time RT-PCR analysis of mRNA levels for *Bnip3* (**B**), *Ctsl* (**C**) and *Map1lc3b* (**E**) in tibialis anterior muscles of untreated (–) and i.p. spermidine-treated (+) wild-type and *col6a1*^−/−^ mice (*n* = 3 or 4; ***, *P* < 0.001; ** *P* < 0.01; *, *P* < 0.05; NS, not significant). 24 h, twenty-four hour-long starvation; Spd IP, spermidine i.p.; Spd PO, spermidine *per os*. WT, wild-type.

Spermidine induces autophagy through modifications at the level of the acetylproteome, thus modifying the expression levels of several genes.^9,10^ In previous studies aimed at mechanistic insight into the autophagic defects of COL6-related myopathies, we have demonstrated that *col6a1*^*−/−*^ muscles display a hyperactivation of the AKT-MTOR pathway and that this signaling defect plays a key role in the autophagy impairment.^8^ AKT kinase negatively regulates the activity of FOXO (forkhead box O) transcription factors by phosphorylation, and decreased levels of phosphorylated AKT represent a permissive condition for FOXO activation.^19,20^ To understand whether the autophagy-inducing effects of spermidine involve the AKT-FOXO axis, we investigated the activation state of AKT and the mRNA levels of several FOXO target genes in tibialis anterior of wild-type and *col6a1*^*−/−*^ mice subjected to i.p. treatment with spermidine at 50 mg/kg for 10 d. Chronic spermidine administration led to decreased AKT phosphorylation, an effect which was particularly strong in *col6a1*^*−/−*^ mice subjected to 24 h starvation (**Fig. 3A**). Real time PCR for *Bnip3* (BCL2/adenovirus E1B 19kDa interacting protein 3), *Ctsl* (cathepsin L), and *Map1lc3b*, 3 autophagy-related genes regulated by the AKT-FOXO axis,^19^ showed significantly increased levels of their transcript in spermidine-treated, 24-h-fasted *col6a1*^*−/−*^ mice (**Fig. 3B to D**). These results indicate that spermidine elicits AKT dephosphorylation, which in turn triggers the translocation of FOXO transcription factors into the nucleus with the consequent increased transcription of FOXO target genes, including autophagy-related genes.

Of note, high-dose spermidine i.p. treatment led to decreased AKT phosphorylation levels in tibialis anterior of wild-type mice even under fed conditions. Yet, AKT phosphorylation was restored when spermidine treatment was followed by 24-h starvation (**Fig. 3A**). This apparently paradoxical finding likely relies upon the stimulatory activity of several FOXO target genes, such as *Sesn3* (sestrin 3) and *Irs1* (insulin receptor substrate 1), on AKT. In turn, active AKT is known to upregulate protein synthesis and to inhibit FOXO activity, as a part of a feedback mechanism that maintains the balance between protein synthesis and protein degradation inside the cells.^21^ In agreement with this concept, tibialis anterior of wild-type mice subjected to prolonged (48 h) fasting displayed significantly higher levels of phosphorylated AKT than those of 24-h-fasted wild-type animals (**Fig. S5A**). At the same time, expression of FOXO target genes was maintained at high levels in these muscles, as found for spermidine-treated and 24-h-fasted wild-type mice (**Fig. 3B-D**; **Fig. S5B-D**). Therefore, the excessive and prolonged stimulus for autophagy induction elicited in wild-type muscles by high-dose spermidine administration in combination with fasting induces a feedback mechanism that restores AKT phosphorylation.

Altogether, these data indicate that spermidine affects AKT activation and enhances autophagy by promoting the transcription of FOXO target genes.

## Discussion

Spermidine is a nontoxic autophagy-inducing natural compound, with an increasing interest for its applications as a nutraceutical tool for therapies. Spermidine counteracts aging and promotes longevity in different species by increasing the basal autophagy levels.^9,10^^,^^22^ Recent work suggests that this polyamine may be beneficial for counteracting several age-related pathologies, such as arterial aging and memory impairment.^23-24^ Moreover, spermidine shows a rapamycin-like effect in promoting the removal of brain protein aggregates and ameliorating the phenotype of a mouse model for TDP-43 proteinopathies, suggesting that it may be a useful route for the therapy of neurodegenerative diseases such as frontotemporal lobar dementia.^25^ Published studies demonstrate the efficacy of spermidine in different models, but no study has ever investigated the effects of this autophagy-inducing compound in muscle diseases.

In this work, we found that spermidine is effective in reactivating autophagy in skeletal muscle and ameliorating the myopathic defects of *col6a1*^*−/−*^ mice, the animal model of UCMD and BM, thus showing for the first time the efficacy of this nutraceutical in a genetically determined muscle disease model. The beneficial effects of spermidine administration in *col6a1*^*−/−*^ mice are associated with its ability to reactivate the autophagic flux, a process which is overtly impaired in this animal model as well as in the corresponding human myopathies.^8^ Indeed, we have previously demonstrated that the muscle defects of *col6a1*^*−/−*^ mice are strictly linked to a failure to maintain a proper autophagic flux in myofibers.^8^ Inefficient autophagy in *Col6a1*-null muscles leads to the progressive accumulation of harmful organelles inside myofibers, which progressively degenerate. However, this autophagy failure is reversible and reactivation of a proper autophagic flux in *col6a1*^*−/−*^ mice, through dietary and pharmacological approaches, leads to a substantial rescue of the myopathic pathology.^5-8^ Here, we investigated the efficacy of spermidine in this well-established myopathic model at different doses and different routes of administration. We observed that high-dose i.p. treatment is very effective in reactivating autophagy, with a significant increase of LC3B lipidation and autophagosome formation. The effects of spermidine on autophagy reactivation in *col6a1*^*−/−*^ muscles were reinforced by 24-h starvation. Even under the fed condition, autophagy flux was induced in spermidine-treated *col6a1*^*−/−*^ mice, as demonstrated by increased levels of RAB7 and GABARAP-II proteins and by accumulation of LC3-II and SQSTM1 in the presence of colchicine cotreatment, and this was nicely paralleled by a conspicuous amelioration of the histology of tibialis anterior.

BM and UCMD patients suffer from respiratory insufficiency due to a loss of functionality of diaphragm,^18^ which is also the most affected muscle in *col6a1*^*−/−*^ mice where it displays marked ultrastructural defects and high incidence of apoptotic myofibers.^16^ Both these aspects were rescued by spermidine. High-dose i.p. treatment showed a remarkable effect on the number of TUNEL-positive nuclei in *col6a1*^*−/−*^ animals, bringing it to similar levels to wild-type control mice. Moreover, ultrastructural analysis showed a marked decrease in the percentage of aberrant mitochondria and several autophagosomes were observed in the proximity of mitochondria in myofibers of *col6a1*^*−/−*^ mice subjected to high-dose i.p. spermidine treatment. Although it is currently not technically feasible to identify the content of these autophagic vacuoles, the major evidence supporting the efficacy of spermidine in inducing autophagy in *col6a1*^*−/−*^ muscles is that, at the difference from untreated conditions,^8^ autophagosomes can be detected in diaphragm of *col6a1*^*−/−*^ mice following spermidine treatment. It is plausible that removal of altered mitochondria via spermidine-induced autophagy decreases the levels of proapoptotic factors released by these organelles, as we previously have observed in *col6a1*^*−/−*^ mice subjected to prolonged starvation or low-protein diet.^8,26^ In addition to this, the antiapoptotic effect of spermidine may also depend on a direct influence of this polyamine on the regulation of programmed cell death or on the crosstalk between the regulatory networks of these 2 processes.^26,27^

Conversely, in wild-type mice, high-dose spermidine administration had some adverse effects, which however were expected. Autophagy induction in spermidine-treated wild-type muscles was promptly detectable even in fed conditions, and it is well known that excessively induced autophagy is detrimental for myofiber homeostasis.^8,28^ The most detrimental known effect of spermidine is due to its capability to inhibit NOS1 (nitric oxid synthase 1 [neuronal]), but pharmacological studies show that the LD_50_ of intraperitoneally injected spermidine is 620 mg/kg,^29^ more than 10 times higher than the highest dose used in the present study. Although we did not observe any overt sign of histological muscle changes in wild-type animals treated with high-dose spermidine, they displayed decreased muscle strength and an increased number of TUNEL-positive myonuclei. These features are typical in skeletal muscle when the autophagy flux is too elevated,^8,28^ but they may also rely on an action of spermidine on multiple aspects of muscle cell biology. In fact, polyamines are able to induce or inhibit programmed cell death based on the environmental conditions and the cell type examined, through direct or indirect effects on molecules or signaling pathways.^26^ It would be interesting to investigate in the future the effect of spermidine on muscle contractile functions. It is possible, for example, that this effect is correlated at least in part with the modulation of ion channels activity by polyamines.^30^

Among the broad range of intracellular targets for spermidine,^9-11^^,^^30^ which may mediate its beneficial effects in *col6a1*^*−/−*^ muscles, we focused on the AKT-MTOR axis. We previously demonstrated that the defective autophagy of *col6a1*^*−/−*^ muscles is characterized by excessive AKT phosphorylation even after a typical proautophagic stimulus, such as 24-h starvation.^8^ This persistent AKT activity causes a constitutive activation of the MTOR pathway and prevents the transcription of several autophagy related genes under the control of FOXO transcription factors.^8,19^ Our data indicate that high-dose i.p. spermidine treatment is capable of normalizing AKT phosphorylation in *col6a1*^*−/−*^ tibialis anterior leading to increased expression of FOXO target genes upon starvation, which is sufficient to induce autophagy in skeletal muscle.^19^ Interestingly, chronically-administered spermidine displayed a robust effect on AKT activity in skeletal muscle. In muscles of wild-type animals, where the AKT pathway is properly working, chronically-administered spermidine led to a significant reduction of AKT phosphorylation under fed conditions, while upon nutrient deprivation AKT phosphorylation was reinstated, an effect that we also observed in wild-type animals subjected to prolonged starvation. This apparently paradoxical effect is likely due to a feedback mechanism, as several transcriptional targets of FOXO are able to upregulate the PI3K-AKT pathway by triggering an increase of the active phosphorylated form of AKT.^21^ In *col6a1*^*−/−*^ animals AKT rephosphorylation is not taking place, and combination of spermidine plus 24 h starvation bring *col6a1*^*−/−*^ muscles to the AKT state observed in muscles of wild-type mice subjected to 24 h starvation but without drug treatment. FOXO transcription factors are regulated by different post-translational modifications, and among these inhibitory acetylation by the histone acetyltransferase EP300 has been thoroughly described.^31^ As spermidine also acts as a histone acetyltransferase inhibitor, and one of its known targets is EP300,^11^ it is plausible that spermidine controls FOXO activity at multiple levels and determines a permissive condition for its activity by decreasing both its phosphorylation by AKT and its acetylation by EP300.

An interesting finding that emerges from our data is that *per os* spermidine administration reactivates autophagy and in parallel has beneficial effects on the phenotype of *col6a1*^*−/−*^ mice. Even if thus far only proven in the *Col6a1*-null animal model, these data are of major interest for prospective therapies as they mean that it is possible to deliver a biologically active concentration of this nutraceutical polyamine in myopathic muscles by oral administration. Although beyond the aim of the present work, it will be interesting in the near future to carry out further analysis, including detailed pharmacokinetic studies of spermidine following oral administration, aimed at devising the optimal regimens able to provide the most effective and enduring beneficial outcomes in the different muscles. For example, it would be interesting to treat animals *per os* with the lowest effective doses of spermidine starting from weaning and throughout their entire life. Furthermore, synergic approaches could be tested using different combinations of spermidine with other nutritional or pharmacological approaches shown to induce autophagy, such as the low-protein diet. Based on our results with spermidine and acute starvation, it is likely that these approaches will show additive effects. A major advantage of spermidine is that it is a natural compound, present at high concentrations in foods such as mushrooms or soybean, and that it can be easily administered.^32^

In conclusion, our data in *col6a1*^*−/−*^ animals point at spermidine as a nutraceutical therapeutic option for COL6-related myopathies and indicate that this approach is valuable for the design of novel treatments, with spermidine alone or in combination with other strategies, and their translation into clinical trials. In perspective, this nutraceutical-based, autophagy-inducing approach could be applied to UCMD and BM therapy, as it has been done for the low-protein diet (ClinicalTrials.gov Identifier NCT01438788), and could also be extended to other inherited muscle diseases involving a defective activity of the autophagy machinery.

## Materials and Methods

### Animals

C57BL/6N mice were obtained from Charles River. *Col6a1*^*+/−*^ mice were backcrossed for at least 8 generations in the inbred C57BL/6N strain. Mice bearing a GFP-LC3 transgene in the C57BL/6J background were obtained from RIKEN BRC (RBRC No. RBRC00806).^33^ GFP-LC3 mice were backcrossed in the C57BL/6N strain and then crossed with *col6a1*^*−/−*^ mice to generate *col6a1*^*−/−*^;GFP-LC3 animals. All mice were housed in a controlled environment with 12 h light/12 h dark cycle and a temperature of 23°C. When maintained in fed conditions, mice were provided with *ad libitum* standard chow (Laboratorio Dottori Piccioni, #52) and water. For starvation experiments, at 9 a.m. mice were individually moved to clean cages without chow but with free access to the water, for 24 h or 48 h. Experiments were carried out on sex- and age-matched mice, ranging from 18 to 24 wk of age. All the animals were sacrificed by cervical dislocation, and the tissues of interest were immediately dissected and fixed. The adopted procedures were approved by the Ethics Committee of the University of Padova and carried out according to all pertinent Italian laws.

### Spermidine treatments

Spermidine (Sigma-Aldrich, S4139) was either dissolved in physiological (NaCl 0.9% w/v) sterile solution or added to drinking water. For the i.p. treatment, each mouse was injected once a d for 10 d with spermidine at 5 mg/kg body weight (low dose) or 50 mg/kg body weight (high dose). Control mice were not injected. Mice were sacrificed within 16 h after the last injection. *Per os* treatment was performed by adding spermidine to the drinking water at a concentration of 3 mM (low dose) or 30 mM (high dose). Mice had free access to water for 30 d and then they were sacrificed. Water with spermidine was replaced every 2 d, in order to avoid excessive deamination of the active compound.^9^ Control mice drank water without addition of spermidine. For starvation experiments, mice were deprived of food during the last day of treatment. For colchicine co-treatment, mice received during the last 2 d of treatment daily i.p. injections with 0.4 mg/kg/day of colchicine (Sigma-Aldrich, C9754).

### Fluorescence microscopy and histology

For the histological analysis, frozen tibialis anterior muscles were cut in 10-μm-thick sections and stained with haematoxylin-eosin (Sigma-Aldrich, 51275 and HT110116) or with a SDH-specific colorimetric substrate (nitro blue tetrazolium, Sigma-Aldrich, N5514), following standard protocols. Images were captured using a Zeiss Axioplan microscope (Carl Zeiss AG, Oberkochen, Germany) equipped with a Leica DC 500 digital camera (Leica Microsystems GmbH, Wetzlar, Germany). For the fluorescent microscopy analysis of GFP-LC3-positive puncta, tibialis anterior was processed as previously described,^33^ stained with Hoechst 33258 (Sigma-Aldrich, B1155) and observed with a Leica SP5 confocal microscope (Leica Microsystems GmbH, Wetzlar, Germany).

### TUNEL assay

Diaphragm muscles were fixed with paraformaldehyde, embedded in paraffin, and cut into 7-μm-thick transverse sections. Nuclei were stained with Hoechst 33258 (Sigma-Aldrich, B1155) and apoptotic nuclei detected by the ApopTag Peroxidase In Situ Apoptosis Detection Kit (Merck Millipore, S7100). Images of the stained sections were captured randomly using a Zeiss Axioplan fluorescence microscope (Carl Zeiss AG, Oberkochen, Germany) equipped with a Leica DC 500 digital camera (Leica Microsystems GmbH, Wetzlar, Germany), and TUNEL-positive nuclei quantified.

### Transmission electron microscopy

Samples were fixed with 1% glutaraldehyde (Sigma-Aldrich, G5882) in 0.2 M sodium cacodylate buffer, pH 7.4. The specimens were washed in distilled water, stained with 2% osmium tetroxide and 0.5% aqueous uranyl acetate, dehydrated in alcohol and embedded in epon resin. Ultrathin (60 nm) sections were cut with a Leica EM UC7 ultramicrotome (Leica Microsystems GmbH, Wetzlar, Germany). Images were acquired from thin sections using a FEI Tecnai 12 transmission electron microscope (FEI Company, Hillsboro, Oregon, USA) equipped with a Veleta CCD digital camera (Olympus Soft Imaging Solutions GmbH, Münster, Germany). For morphometric analysis, mitochondria were classified as “aberrant” when they displayed at least one of the following abnormalities: swollen morphology, faintly stained inner matrix, dilated cristae and/or overtly decreased number of cristae.

### Western blotting

Protein extraction from muscles and western blot analysis were performed as previously described.^34^ Primary antibodies against the following proteins were used: AKT (Cell Signaling Technology, 9272), phospho-AKT (Ser473; Cell Signaling Technology, 4058), LC3B (Thermo Scientific, PA1–16930), GAPDH (Millipore, MAB374), SQSTM1/p62 (Santa Cruz Biotechnology, [H-290], sc-25575), RAB7 (Abcam, [RAB7–117], ab50533), GABARAP (Santa Cruz Biotechnology, [FL-117], sc-28938). Densitometric quantification was performed by ImageJ software (NIH, Bethesda, Maryland, USA).^35^

### Real time RT-PCR

Whole tibialis anterior muscles were blended, and RNA extraction performed using TRIzol reagent (Life Technologies, 15596–018) in accordance with the manufacturer's instructions. 500 ng of total RNA were retrotranscribed using the SuperScript III kit (Life Technologies, 18080–051) following manufacturer's instructions. Quantitative PCR was performed on a RotorGeneQ instrument (Qiagen, Venlo, Netherlands), using specific primers and CyBr green contaning mastermix (Qiagen, 204076). Primer sequences were described elsewhere.^8,19^

### Tetanic force measurements

In vivo determination of force and contraction kinetics of gastrocnemius muscle was carried out as previously described.^36^ Briefly, mice were anesthetized and contraction elicited by stimulation of the sciatic nerve with multistranded steel wires (Cooner Wire, Chatsworth, CA, USA) implanted on either sides of the nerve. In order to avoid recruitment of the ankle dorsal flexors, and therefore significant reduction of torque, the common peroneal nerve was cut. Torque production of the stimulated plantar flexors was measured using a muscle lever system (Aurora Scientific Corporation, Aurora, Ontario, Canada). The force-frequency curves were determined by stepwise increasing stimulation frequency, pausing at least 30 s between stimuli to avoid effects due to fatigue. Tetanic contraction was reached for stimulations equal to or greater than 100 Hz, and force values obtained in these settings were compared between the different conditions as they represent the maximum contraction muscle can sustain.

### Statistical analysis

All data are expressed as mean ± s.e.m. Statistical analysis of data was carried out using the Student *t* test, except for the electron microscope morphometric analysis where the Wilcoxon rank sum test was used. *P* < 0.05 was considered as a significant difference.
