# Supplementary figures and images for: Reactivation of autophagy by spermidine ameliorates the myopathic defects of collagen VI-null mice
Source: Autophagy. 2015 Nov 13;11(12):2142–52. doi: 10.1080/15548627.2015.1108508 (PMC4835186; doi:10.1080/15548627.2015.1108508)

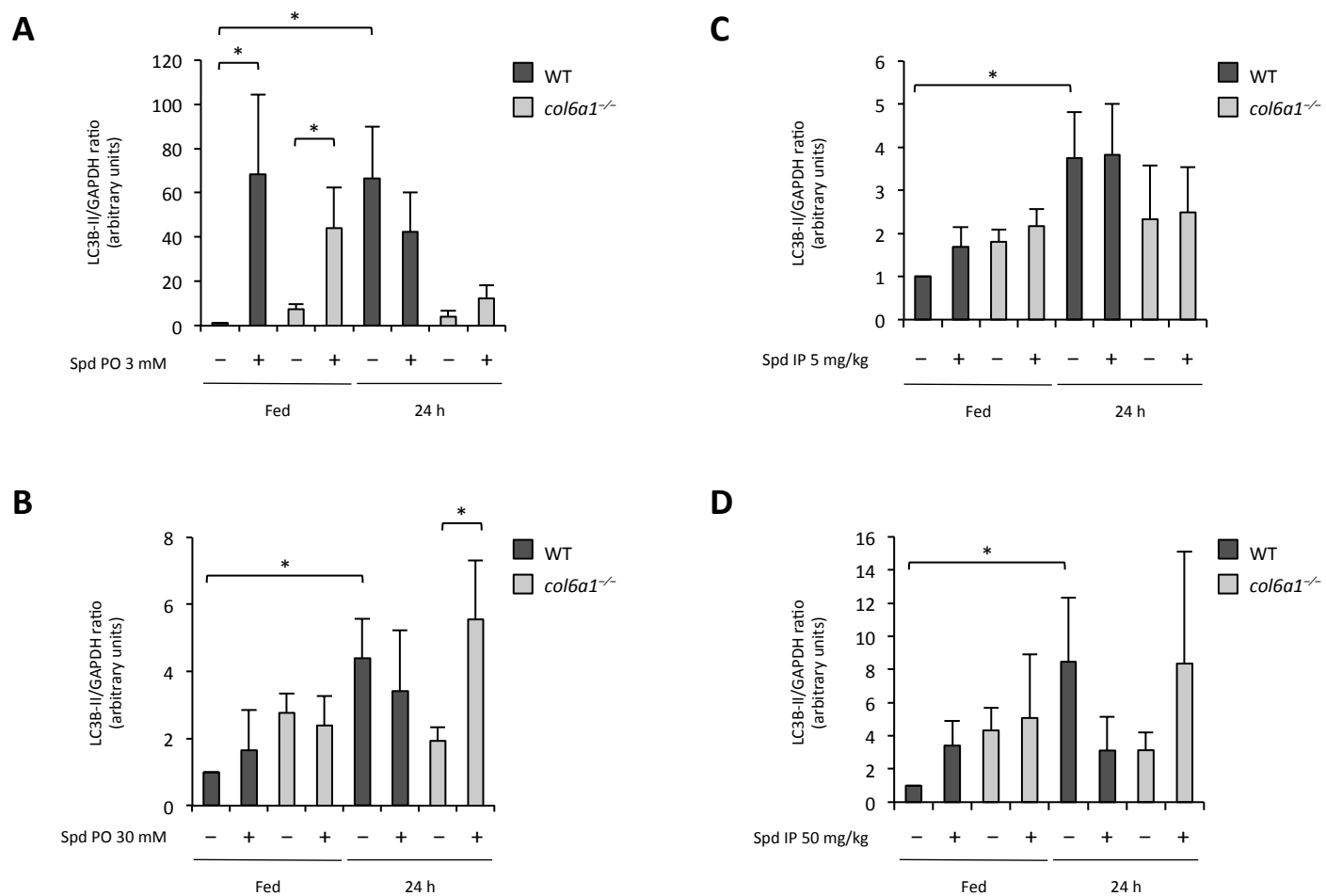

**Figure S1**

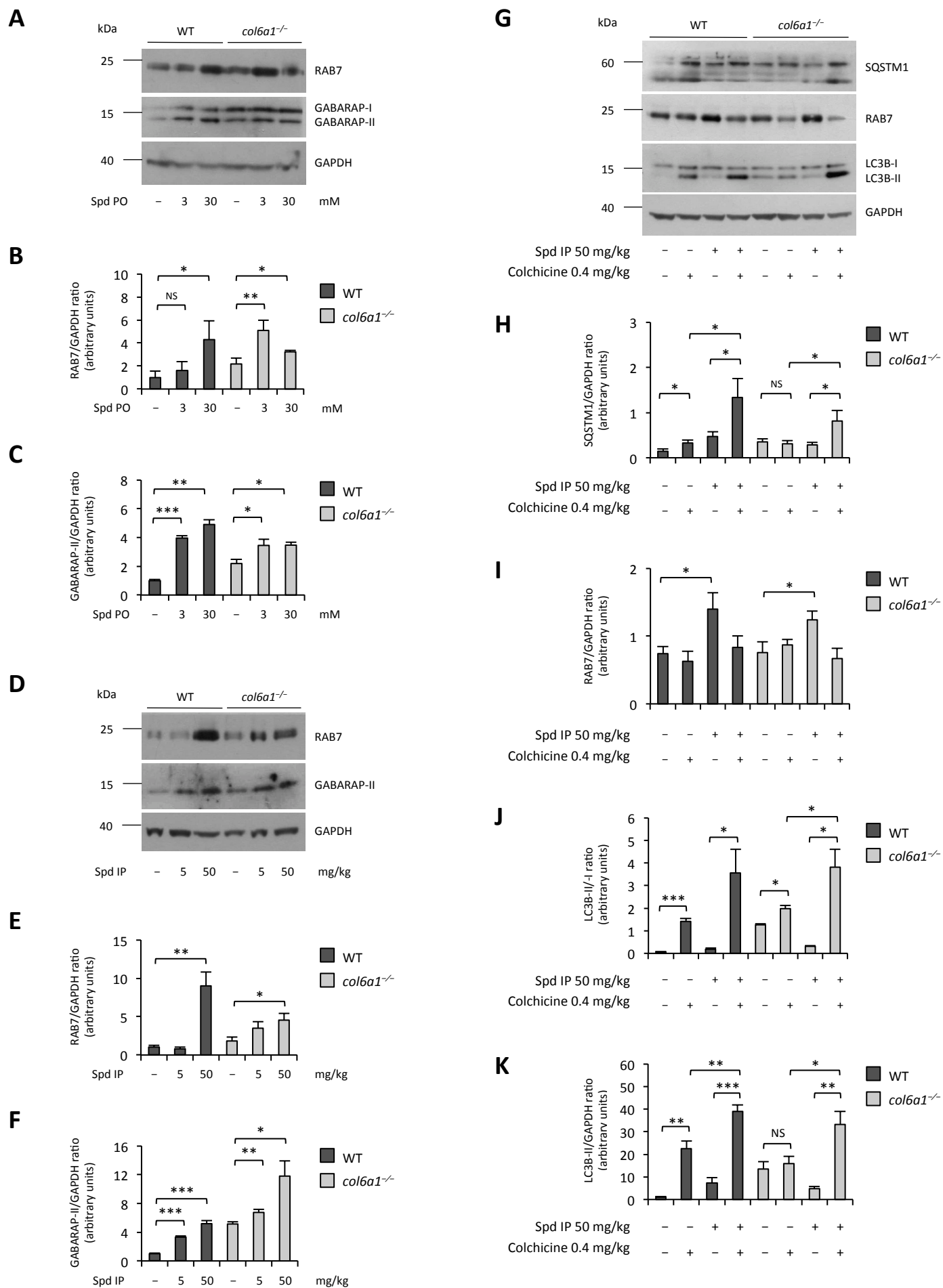

**Figure S2**

**A**

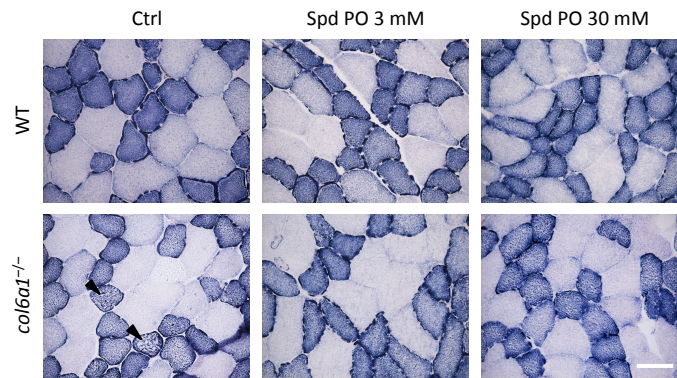

**B**

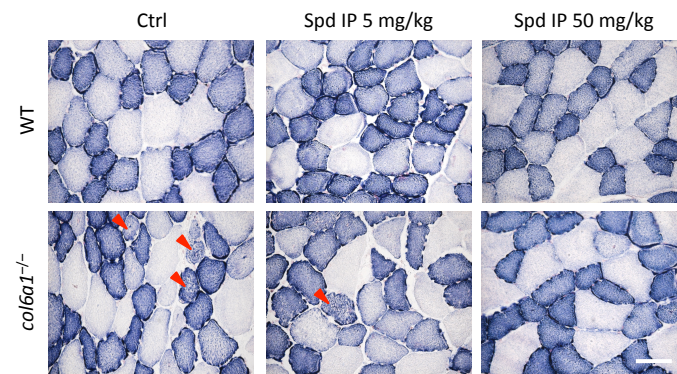

**Figure S3**

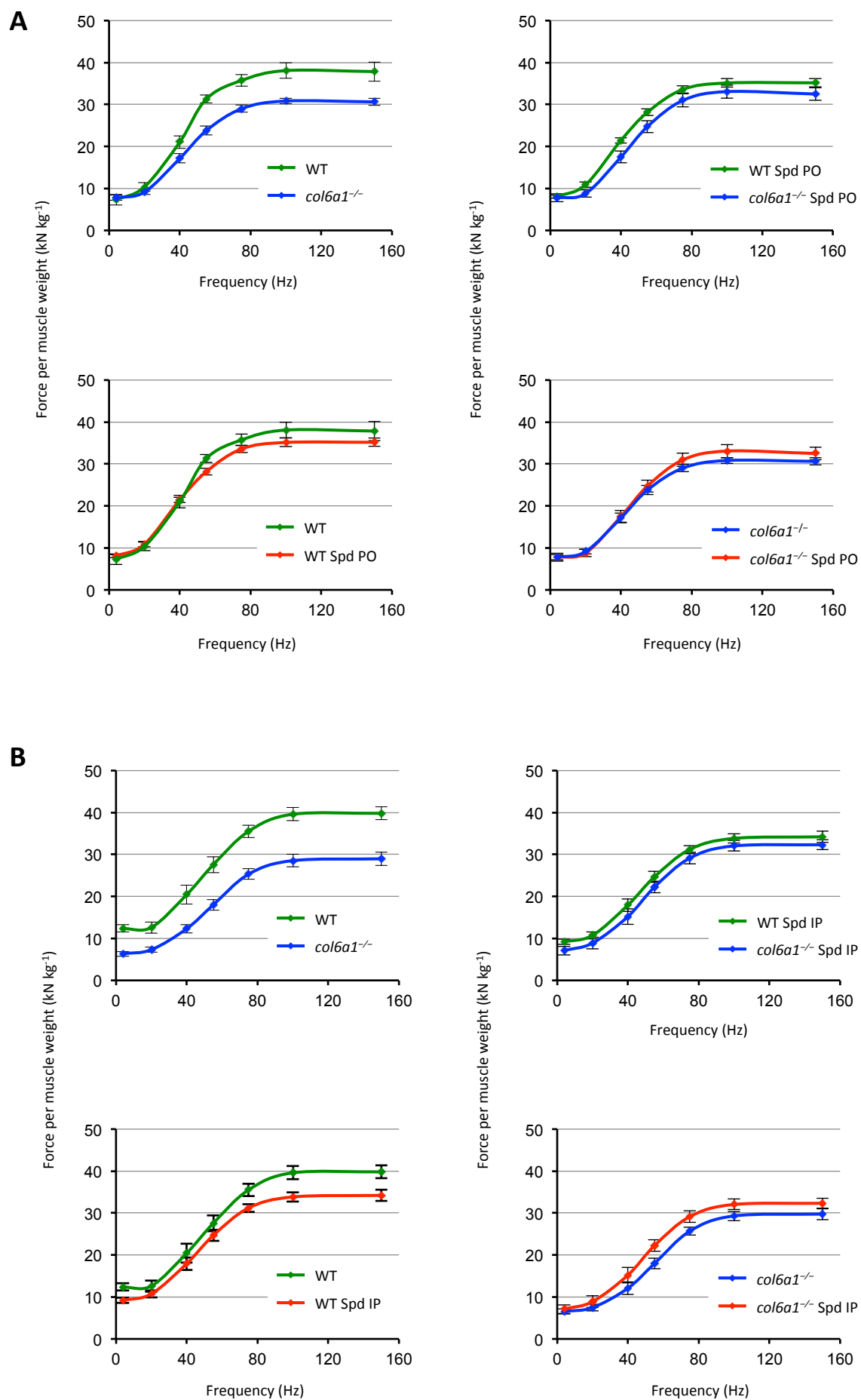

**Figure S4**

**A**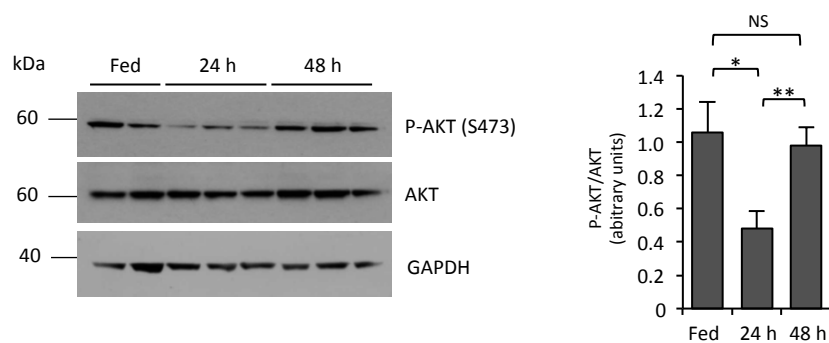**B**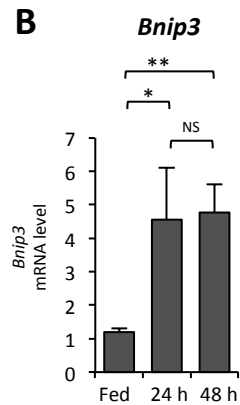**C**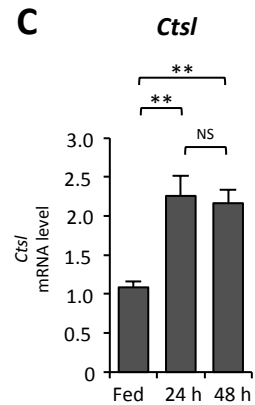**D**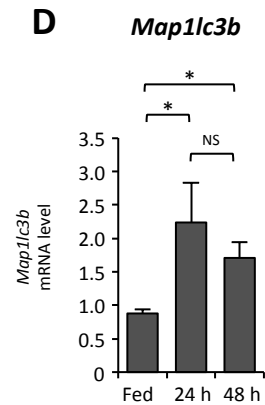**Figure S5**

Supplement: 1108508_Supplemental_Material.zip [file kaup-11-12-1108508-s001.zip › Supplemental Figures.pdf]
