# Supplementary material for: Reactivation of autophagy by spermidine ameliorates the myopathic defects of collagen VI-null mice
Source: Autophagy. 2015 Nov 13;11(12):2142–52. doi: 10.1080/15548627.2015.1108508 (PMC4835186; doi:10.1080/15548627.2015.1108508)
Supplement: 1108508_Supplemental_Material.zip [file kaup-11-12-1108508-s001.zip › 1108508_Supplemental Material.doc]

**Supplementary material**

**Reactivation of autophagy by spermidine ameliorates the myopathic defects of collagen VI null mice**

Martina Chrisam^1^, Marinella Pirozzi^2^, Silvia Castagnaro^1^, Bert Blaauw^3^, Roman Polishchuck^2^, Francesco Cecconi^4,5,6^, Paolo Grumati^1,7*^ and Paolo Bonaldo^1*^

^1^Department of Molecular Medicine, University of Padova, Padova, Italy.

^2^Telethon Institute of Genetic and Medicine (TIGEM), Napoli, Italy.

^3^Venetian Institute of Molecular Medicine (VIMM), Padova, Italy.

^4^IRCCS Fondazione Santa Lucia, 00143 Rome, Italy

^5^Department of Biology, University of Rome Tor Vergata, 00133 Rome, Italy

^6^Danish Cancer Society Research Center, 2100 Copenhagen, Denmark

^7^Institute of Biochemistry II, Goethe University, Frankfurt am Main, Germany.

*Correspondence should be addressed to Paolo Bonaldo ([bonaldo@bio.unipd.it](mailto:bonaldo@bio.unipd.it)) or Paolo Grumati ([paolo.grumati@unipd.it](mailto:paolo.grumati@unipd.it))

**Table S1.** Summary of the different treatment protocols used in this study.

| ***Treatment*** | ***Dose*** | ***Duration of treatment*** | ***Food starvation*** | ***Abbreviation*** |
| --- | --- | --- | --- | --- |
| Low dose spermidine by i.p. injection, fed | 5 mg/kg | 10 days | no | Spd IP 5  Fed |
| Low dose spermidine by i.p. injection, fasting | 5 mg/kg | 10 days | 24 h | Spd IP 5  24 h |
| High dose spermidine by i.p. injection, fed | 50 mg/kg | 10 days | no | Spd IP 50  Fed |
| High dose spermidine by i.p. injection, fasting | 50 mg/kg | 10 days | 24 h | Spd IP 50  24 h |
| Low dose spermidine in drinking water, fed | 3 mM | 30 days | no | Spd PO 3  Fed |
| Low dose spermidine in drinking water, fasted | 3 mM | 30 days | 24 h | Spd PO 3  24 h |
| High dose spermidine in drinking water, fed | 30 mM | 30 days | no | Spd PO 30  Fed |
| High dose spermidine in drinking water, fasted | 30 mM | 30 days | 24 h | Spd PO 30  24 h |
| No treatment, fed (control) | – | – | no | Ctrl  Fed |
| No treatment, fasted (control) | – | – | 24 h | Ctrl  24 h |

**Supplementary Figure Legends**

**Figure S1.** Densitometric quantifications of LC3B-II *vs* GAPDH, as determined by 3 independent western blot experiments, in protein extracts from tibialis anterior muscles of untreated (–), *per os* spermidine-treated (+; **A, B**) or i.p. spermidine-treated (+; **C, D**) wild-type and *col6a1*^–/–^ mice, under fed conditions or following 24-h starvation (*n* = 3 or 4; *, *P* < 0.05). 24 h, 24-h starvation; Spd IP, spermidine i.p.; Spd PO, spermidine *per os*; WT, wild type.

**Figure S2.** (**A to F**) Western blot analysis of RAB7 and GABARAP protein levels in protein extracts from tibialis anterior muscles of untreated (–), *per os* spermidine-treated (+; **A**) or i.p. spermidine-treated (+; **D**) treated wild-type and *col6a1*^–/–^ mice under fed conditions. Densitometric analysis, as determined by 3 independent western blot experiments, for RAB7 *vs* GAPDH (**B, E**) or lipidated GABARAP (GABARAP-II) *vs* GAPDH (**C, F**) are shown below the representative immunoblotting images (*n* = 3 or 4; *, *P* < 0.05; ** *P* < 0.01; *** *P* < 0.001; NS, not significant). WT, wild type. (**G to K**) Western blot analysis of SQSTM1, RAB7 and LC3B in protein extracts from tibialis anterior muscles of untreated (–) or i.p. spermidine-treated (+) wild-type and *col6a1*^–/–^ mice in the presence (+) or absence (–) of colchicine. Representative immunoblots (**G**) and densitometric quantifications, as determined by at least 3 independent western blot experiments, of SQSTM1 *vs* GAPDH (H), RAB7 *vs* GAPDH (I), LC3B-II *vs* LC3B-I (**J**) and LC3B-II *vs* GAPDH (**K**) are shown (*n* = 3 to 4; *, *P* < 0.05; ** *P* < 0.01; *** *P* < 0.001; NS, not significant). Spd IP, spermidine i.p.; WT, wild type.

**Figure S3.** SDH staining of cross sections of tibialis anterior muscles from untreated (Ctrl) and and *per os* (**A**) or i.p. (**B**) spermidine-treated wild-type and *col6a1^–/–^* mice. Red arrowheads point at some myofibers with markedly altered mitochondrial labeling. Scale bar: 50 μm. Spd IP, spermidine i.p.; Spd PO, spermidine *per os*. WT, wild type.

**Figure S4.** Normalized force measurements of gastrocnemius muscle of wild-type and *col6a1^–/–^* mice under untreated conditions and following spermidine *per os* (Spd PO) treatment at 30 mM or spermidine i.p. (Spd IP) treatment at 50 mg/kg. Whole curves for different stimulation frequencies are shown. Tetanus was reached at a stimulation frequency of 100 Hz. WT, wild type.

**Figure S5.** Effects of prolonged starvation on the AKT-FOXO axis regulation. (**A**) Western blot analysis of AKT phosphorylation in protein extracts from tibialis anterior muscles from wild type animals fasted for different periods of time. Densitometric quantifications of P-AKT *vs* AKT, as determined by 3 independent western blot experiments, are shown on the right panel (*n* = 3 to 4; *, *P* < 0.05; ** *P* < 0.01; NS, not significant). (**B** to **D**) Real-time RT-PCR analysis of mRNA levels for *Bnip3* (**B**), *Ctsl* (**C**) and *Map1lc3b* (**D**) in tibialis anterior muscles of wild-type animals fasted for different periods of time (*n* = 3; *, *P* < 0.05; ** *P* < 0.01; NS, not significant). 24 h, 24-h starvation; 48 h, 48-h starvation; WT, wild type.
